# Physiotherapy in Spinocerebellar Ataxia Following COVID‐19: A Biomechanical and Biopsychosocial Case Report

**DOI:** 10.1002/pri.70122

**Published:** 2025-11-20

**Authors:** Luiz Humberto Figueiredo Monteiro, Isabela Natalia de Souza Rêgo, Ana Beatriz Souza da Conceição, Hugo Miranda de Souza Coroa, Brenno Ribeiro Braz, Suellen Alessandra Soares de Moraes

**Affiliations:** ^1^ Instutite of Health Sciences Federal University of Pará (UFPA) Belém Pará Brazil; ^2^ Metropolitan Urgency and Emergency Hospital University of Pará State (UEPA) Belém Pará Brazil

**Keywords:** COVID‐19, postural balance, spinocerebellar ataxia

## Abstract

**Background and Purpose:**

Spinocerebellar ataxia (SCA) is a progressive neurodegenerative disorder characterized by impaired postural control, coordination deficits, and functional limitations. Despite evidence supporting physiotherapy interventions, standardized rehabilitation protocols remain scarce. This case report explores the effects of an evidence‐based physiotherapy program on functionality, baropodometric and stabilometric parameters, and quality of life (QoL) in a patient with SCA.

**Methods:**

Case Presentation—A 56‐year‐old male with clinically diagnosed SCA and a history of COVID‐19 infection preceding symptom onset underwent 30 physiotherapy sessions focused on muscle strengthening, balance training, and coordination exercises. Functional and quantitative assessments included the Timed Up and Go (TUG) test, Romberg test, Dizziness Handicap Inventory (DHI), and baropodometric and stabilometric analyses.

**Results:**

The patient reported subjective improvement in dizziness but showed no significant changes in balance or mobility. Post‐intervention baropodometric results revealed normalization of bilateral contact surface with eyes closed and an improvement in the plantar arch index of the left foot with eyes open. However, stabilometric assessments indicated an increase in the Center of Pressure (COP) area and mean oscillation velocity in both visual conditions, suggesting a decline in postural stability. The DHI score improved by nearly 50%, particularly in the physical domain.

**Discussion:**

This case report highlights the role of physiotherapy in managing functional impairments in SCA, particularly dizziness and foot biomechanics. However, the limited impact on postural stability suggests the need for complementary rehabilitation strategies, such as vestibular rehabilitation and intensive functional training. Integrating baropodometry and stabilometry into routine assessments may facilitate individualized treatment planning and better track disease progression. These findings emphasize the importance of a multidisciplinary, long‐term rehabilitation approach to optimize functional outcomes and enhance QoL in individuals with SCA.

## 1 Introduction

Pierre Marie first described autosomal dominant cerebellar ataxia (ADCA) in 1893, and Harding's classification (ADCA I–III) was widely used until genetic advances led to a mutation‐based classification, now termed spinocerebellar ataxia (SCA), encompassing 48 recognized subtypes. SCA prevalence ranges from 0.9 to 3 per 100,000 individuals, varying by subtype and region. Globally, SCA1, SCA3, and SCA6 are the most common, with SCA3 (Machado–Joseph disease) being the most prevalent in Brazil (Sharma et al. 2022; Yap et al. 2022; Martins et al. 2013).

SCAs are autosomal dominant, progressive disorders characterized by balance and coordination loss, often with dysarthria and other neurological deficits (Klockgether et al. 2019). Symptoms typically emerge in middle age, and as the disease advances, motor impairment and muscle weakening significantly reduce the quality of life (QoL), limiting activities and promoting social isolation. Additionally, severe dysarthria, fall risk, and eventual immobility further deteriorate physical and psychological well‐being (Martins et al. 2013; Correia et al. 2023).

Moreover, balance can be objectively assessed through stabilometric analysis using force platforms, enabling the evaluation of plantar pressure distribution by mapping pressure across different foot regions under both static and dynamic conditions. These quantitative assessment tools are critical for tracking disease progression and providing objective data for clinical decision‐making (Fullin et al. 2022; Zwergal et al. 2020; Sobanska et al. 2024).

Despite advances in neurogenetic diagnostics, including next‐generation sequencing, many SCA patients lack a definitive molecular diagnosis due to clinical and genetic heterogeneity. Mutations and noncoding repeat expansions, such as those in the FGF14 gene (SCA27B), complicate genetic identification through conventional methods. This lack of molecular characterization hinders personalized treatment development and reduces prognostic accuracy (Pellerin et al. 2024).

Recent evidence suggests that SARS‐CoV‐2 infection may accelerate the progression of neurodegenerative diseases (Velázquez‐Pérez et al. 2024). Although post‐COVID‐19 ataxia has been reported in a few cases, especially among children (Takao et al. 2023), its association with genetically determined ataxias such as SCA remains rare and poorly documented in the literature (Singh et al. 2021). This case contributes to addressing this gap by documenting a temporal link between COVID‐19 infection and the onset of progressive cerebellar symptoms.

Beyond this clinical relevance, the present case also stands out for its therapeutic approach. The use of baropodometric and stabilometric tools to quantitatively monitor postural control—integrated with a biopsychosocial framework guided by the International Classification of Functioning, Disability, and Health (ICF)—provided a comprehensive, individualized rehabilitation strategy. These elements add methodological originality and practical relevance to this report.

In this context, this case report aims to analyze the effects of an evidence‐based physiotherapy intervention, focused on functionality, balance, and QoL in a patient with SCA.

## 2 Methods

This case report was developed in accordance with the CARE (Case Report) guidelines to ensure transparency, completeness, and methodological rigor in clinical case reporting. Additionally, it was approved by the Human Research Ethics Committee of the Federal University of Pará (Protocol number: 46775421.0.0000.0018), in compliance with Resolution 510/16 of the National Health Council of Brazil.

### 2.1 Case Presentation

This case stands out due to its multifactorial nature, particularly the temporal proximity between COVID‐19 infection and the onset of ataxia symptoms—an association still scarcely explored in the literature. Moreover, it incorporates objective biomechanical assessments (baropodometry and stabilometry) alongside a biopsychosocial evaluation model guided by the International Classification of Functioning, Disability, and Health (ICF). This integrative and evidence‐based physiotherapy approach reinforces the relevance of individualized monitoring and highlights tools that remain underutilized in clinical practice for the management of SCA.

The patient is a 56‐year‐old male, married, former security guard, residing in Belém, Pará, Brazil, who sought physiotherapeutic care due to progressive balance impairment, frequent falls, and walking difficulties. His symptoms first manifested in June 2020, coinciding with a COVID‐19 infection, and he received a clinical diagnosis of SCA in 2021, without genetic confirmation due to the unavailability of testing in the public healthcare system.

Neurological examination revealed an ataxic gait with rightward deviation, reduced dissociation between the shoulder and pelvic girdles, and moderate dysarthria. Additionally, mild rigidity, hypertonia in the hip flexors, knee flexors, and dorsiflexors, as well as an absence of postural sensation in the halluces, were observed. Neurological tests indicated bilateral dysmetria in the finger‐to‐nose and heel‐to‐shin tests, along with hyporeflexia in the deep tendon reflexes. Muscle strength, assessed using the Medical Research Council (MRC) scale, was grade 5 in the upper limbs, grade 4 in hip, knee, and ankle flexion/extension, and grade 3 in hip abduction/adduction. The head impulse test (HIT) identified saccadic movements in the left eye, suggesting central vestibular impairment. The patient reported a family history of Parkinson's disease and Alzheimer's disease and was taking antidepressants and gastric protectors.

**FIGURE 1:**
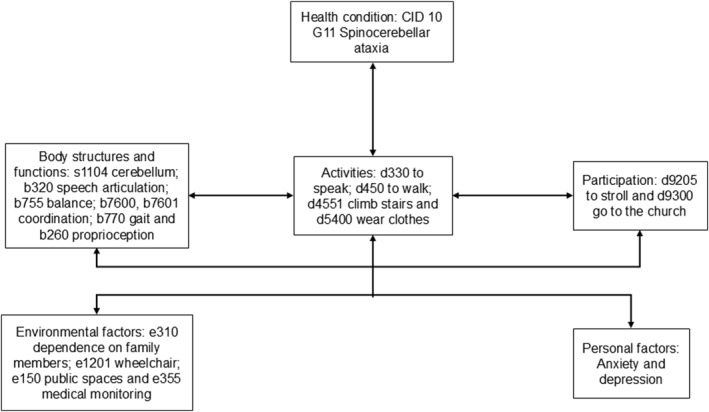
Biopsychosocial analysis following the International Classification of Functioning, Disability, and Health (ICF).

A biopsychosocial assessment was conducted using the International Classification of Functioning, Disability, and Health (ICF) framework, as illustrated in Figure 1. The patient exhibited significant functional limitations, particularly in activities requiring postural control and gait stability. Given the high risk of falls and progressive mobility decline, the use of assistive devices, such as a walker or wheelchair, was deemed necessary to preserve autonomy. From a psychosocial perspective, the patient displayed mild depressive symptoms, exacerbated by reduced social participation and increased dependence on caregivers for daily activities. Nevertheless, strong family support played a pivotal role in ensuring treatment adherence. The biopsychosocial model informed the development of personalized therapeutic strategies, emphasizing functional training, psychological adaptation, and social reintegration.

Following the initial assessment, an evidence‐based physiotherapeutic intervention protocol was designed, comprising 30 sessions, conducted twice per week and focused on muscle strengthening, balance training, and coordination exercises. Before rehabilitation, functional and quantitative assessments were performed, including the Timed Up and Go (TUG), Romberg Test, and Dizziness Handicap Inventory (DHI), to evaluate functional impairment and quality of life. Baropodometric and stabilometric analyses were conducted using the BaroScan force platform (Londrina, PR, Brazil) and BaroSys software with a sampling frequency of 100 Hz. During testing, the patient remained barefoot, in an orthostatic position, for 30 s, first with eyes open (EO) while fixating on a point 2 m away, followed by 1 min of rest, and then with eyes closed (EC). The therapeutic plan incorporated progressive strategies aimed at optimizing functional adaptation, as detailed in Table 1.

**TABLE 1:** Description of physiotherapy interventions, resources, and functional activities implemented for the patient.

Sessions	Physiotherapy intervention
1st–10th	1 Warm‐up: 10‐min treadmill walk. 2 Stretching: Cervical spine, upper and lower limbs. 3 Proprioceptive neuromuscular Facilitation (PNF): Active, active‐assisted, and passive lumbar‐pelvic exercises. 4 Gait training: With and without obstacles, using parallel bars and a walker. 5 Step‐up and down exercise: With ball throwing (3 sets of 10 repetitions). 6 Lumbar‐pelvic dissociation training: 1 set of 10 repetitions. 7 Hip flexion with manual resistance. 8 Bridge exercise: With 1 kg ball passing under the hip.
11th–20th	1. Dual‐task treadmill training. 2. Resistance training: Gastrocnemius, using a yellow elastic band in the supine position (3 sets of 15 repetitions). 3. Bridge exercise: With a ball between the knees (2 sets of 8 repetitions). 4. Flexibility training: Using a ball (2 sets of 10 repetitions). 5. Balance training: Lateral shifts on a balance board while holding a ball at shoulder flexion and elbow extension. 6. Posterior chain stretch: With a yellow elastic band (3 sets of 30 s). 7. Gluteus medius stretching: Assisted by the therapist (3 sets of 15 repetitions). 8. Gait training: Walking over obstacles and 100‐m free walking. 9. Resistance training: Hip abduction with a blue elastic band, combined with functional upper limb reach training using cones. 10. Balance training: Kneeling with trunk rotation (3 sets of 10 repetitions) and semi‐kneeling balance training (3 sets of 10 repetitions). 11. Sit‐to‐stand training: With a blue elastic band around the knees while raising the upper limbs above the head.
21st–30th	1 Warm‐up: 10 min on a treadmill with a 3‐degree incline. 2 Active posterior chain stretching: Assisted by an elastic band, with hip flexion and extended knees (3 sets of 10 repetitions per limb). 3 Bridge exercise: Combined with hip abduction using a blue elastic band (3 sets of 10 repetitions). 4 Trunk extension training: In a prone position on a Swiss ball (3 sets of 10 repetitions). 5 Sit‐to‐stand training: On a platform (3 sets of 12 repetitions). 6 Gait training: On parallel bars over an unstable surface (3 min) and walking 100 m with a walker.

After a detailed explanation of the study, the patient provided written informed consent before initiating the intervention. No adverse effects or discomfort were reported during the 30 physiotherapy sessions, supporting the safety and tolerability of the intervention protocol.

## 3 Results

After completing 30 physiotherapy sessions, the patient reported a subjective improvement in dizziness but no perceived changes in balance or mobility. Objective assessments revealed normalization of the bilateral contact surface with EC and an improvement in the left foot’s plantar arch index with EO. Additionally, there was an increase in the area and mean oscillation speed of the center of pressure (COP) under both visual conditions. Furthermore, a post‐intervention reduction in the length surface function (LSF) was observed in both EC and EO conditions. Neurological reassessment after the intervention revealed no changes in lower limb hypertonia, postural sensation in the halluces, or dysmetria in the upper and lower limbs.

**FIGURE 2:**
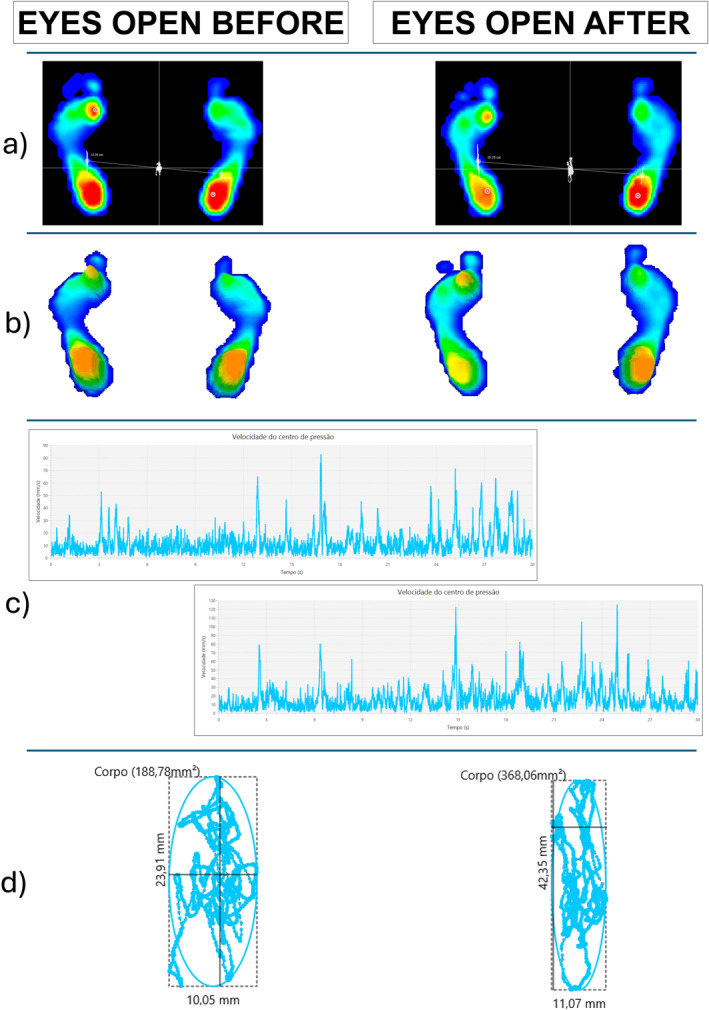
Graphical summary of baropodometric and stabilometric changes with eyes open pre‐ and post‐intervention. (a) 2D plantar pressure map—distribution of plantar pressure before and after the intervention. (b) 3D plantar pressure map—representation of plantar pressure peaks in three dimensions. (c) Velocity graph of the Center of Pressure (COP)—the *x*‐axis represents velocity (mm/s), and the *y*‐axis represents time (s). (d) COP displacement and ellipse representation—the ellipse represents the COP area, while the irregular line indicates COP displacement over time.

**FIGURE 3:**
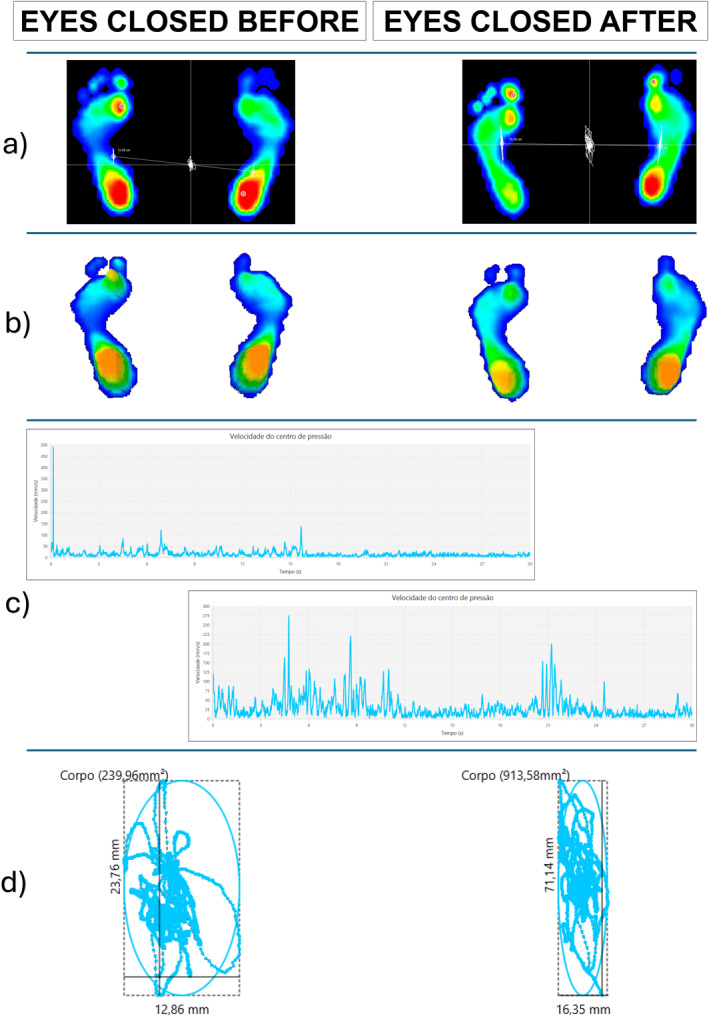
Graphical summary of baropodometric and stabilometric changes with eyes closed pre‐ and post‐intervention. (a) 2D plantar pressure map—distribution of plantar pressure before and after the intervention. (b) 3D plantar pressure map—representation of plantar pressure peaks in three dimensions. (c) Velocity graph of the Center of Pressure (COP)—the *x*‐axis represents velocity (mm/s), and the *y*‐axis represents time (s). (d) COP displacement and ellipse representation—the ellipse represents the COP area, while the irregular line indicates COP displacement over time.

Detailed outcomes of the baropodometric and stabilometric variables are presented in Tables 2 and 3 and illustrated in Figures 2 and 3.

**TABLE 2:** Baropodometric results before and after physiotherapy intervention with reference values for each variable in healthy individuals.

Variable	EO before		EO after		RV		EC before		EC after		RV	
	Left	Right	Left	Right	Left max–min	Right max–min	Left	Right	Left	Right	Left max–min	Right max–min
Contact surface (%)	51.01	48.99	↓50.54	↑49.46	45.65–48.70	51.30–54.35	49.17	50.83	↓48.74	↑51.26	46.15–49	51–53.85
Maximum pressure (Kpa)	205.23	266.38	↓117	↑294.97	57.32–71.52	53.27–65.87	186.27	270.28	↑203.12	↓162.66	58.09–68.64	52.11–64.55
Mean pressure (Kpa)	44.72	53.74	↓37.82	↓49.23	22.96–27.89	22.79–27.32	43.10	52	↓37.27	↓43.95	22.27–26.49	21.94–25.70
Plantar arch index (%)	33	24	↓25	↑31	8.57–25.27	12.98–24.95	23	26	↑25	↑29	9.05–24.48	13.44–24.71

*Note:* ↓ (down) and ↑ (up) arrows indicate whether a variable decreased or increased after the intervention.

Abbreviations: EC, eyes closed; EO, eyes open; Max, maximum; Min, minimum; RV, reference values (De Blasiis et al. 2023).

**TABLE 3:** Stabilometric results before and after physiotherapy intervention.

Variable	Initial assessment		Final assessment		RV	
	EO	EC	EO	EC	EO	EC
COP area (mm^2^)	188.78	239.96	↑368.06	↑913.58	23.50–41.69	13.17–29.22
Length surface function (mm^−1^)	1.88	1.82	↓1.35	↓0.95	3.80–7.10	5.28–9.68
Mean velocity of the COP (mm/s)	11.81	14.6	↑16.62	↑28.91	3.13–3.97	3–3.93

*Note:* ↓ (down) and ↑ (up) arrows indicate whether a variable decreased or increased after the intervention.

Abbreviations: COP, center of pressure; EC, eyes closed; EO, eyes open; RV, reference values (De Blasiis et al. 2023).

In Figure 3c, during the EC condition before the intervention, the participant exhibited a sudden spike in velocity within the first 2 seconds, reaching 500 mm/s, followed by a stabilization phase, where velocity remained below 150 mm/s for the remainder of the trial.

Additionally, the TUG test time increased by 8.21 s, while the Romberg test showed no changes. Conversely, the DHI score improved, with reductions observed across all three domains. Notably, the physical domain demonstrated nearly a 50% improvement in post‐intervention.

## 4 Discussion

This case report shows the effects of an evidence‐based physiotherapy intervention in an individual with SCA, highlighting functional, baropodometric, stabilometric, and quality of life outcomes. Its uniqueness lies in the combination of clinical and contextual factors—particularly the rare temporal association between symptom onset and a prior COVID‐19 infection, the integration of quantitative biomechanical tools for individualized monitoring, and the structured application of the ICF to guide a biopsychosocial rehabilitation plan. These components, seldom reported together in the context of SCA, offer original insights into patient‐centered neurorehabilitation strategies and support the scientific and clinical relevance of presenting this case in the format of a detailed single‐subject report.

The normalization of the plantar arch and contact surface may result from extrinsic foot muscle activation and strengthening, facilitated by targeted exercises such as gastrocnemius resistance training, bridge exercises, and gait activities on unstable surfaces, with and without obstacles, walker‐assisted gait, and lateral movements on a balance board. Additionally, these improvements may stem from the partial preservation of motor learning mechanisms and neural plasticity within the cerebellar circuit (Rodríguez‐Díaz et al. 2018).

In addition, studies have demonstrated that functional exercises, gait training, and foot muscle strengthening improve baropodometric variables, including plantar arch index and plantar pressure distribution, in individuals with diabetic neuropathy (Monteiro et al. 2023), recreational runners (Da Silva Neto et al. 2021), and elderly women (Kim and Park 2015).

The stabilometric results indicated an increase in the oscillation area and mean COP velocity under both visual conditions, suggesting a decline in postural stability following the intervention. This deterioration may be attributed to the natural progression of SCA (Artigas et al. 2013). Moreover, the patient initially presented with gait alterations, a symptom associated with faster disease progression, as suggested by studies linking gait impairments to accelerated SCA progression, particularly in subtypes such as SCA6 (Luo et al. 2017).

Regarding the post‐intervention reduction in LSF, no prior studies have reported this variable in SCA patients. We hypothesize that this reduction reflects a compensatory neuromuscular response aimed at enhancing body stability, albeit ineffectively, further reinforcing the progressive decline characteristic of the disease.

Our hypothesis is supported by De Blasiis et al. (2023), who associate LSF with postural stability. The authors observed that LSF varies according to visual conditions, with a negative correlation between LSF and COP area in the EO condition, suggesting that greater LSF is linked to better postural control, as reflected in a smaller oscillation area. In contrast, in the EC condition, LSF does not correlate with other baropodometric and stabilometric variables, indicating a more independent behavior.

Consistent with a study (Elyoseph et al. 2024) that reported high DHI scores in SCA patients, we also observed elevated scores, with the physical domain improving from 26/28 to 14/28 post‐intervention, a nearly 50% reduction in dizziness perception related to physical function. Additionally, previous studies (Elyoseph et al. 2024; Zwergal et al. 2020; Zeigelboim et al. 2018) have reported vestibular symptoms in SCA patients, including dizziness and saccadic movements, similar to this case.

The results obtained, such as the increased TUG time, the evident rise in body sway area observed through stabilometric analysis, and the persistence of neurological signs—such as hypertonia and dysmetria—align with the progressive nature of SCA. While physiotherapy interventions can alleviate symptoms, they are unable to halt disease progression, leaving patients at high risk of falls and facing challenges in maintaining home‐based exercise programs (Martins et al. 2013; Artigas et al. 2013).

Through this case report and database analysis, we identified a lack of standardized rehabilitation protocols for individuals with SCA. This highlights the importance of quantitative tools, such as baropodometry and stabilometry, in the objective monitoring of SCA progression for both research and clinical practice (Marquer et al. 2014). However, these technologies remain underutilized in clinical settings. We recommend future research focused on developing standardized rehabilitation protocols, further characterizing individuals with SCA through baropodometric parameters, and integrating quantitative assessments to enhance therapeutic efficacy and long‐term disease monitoring.

Furthermore, within the biopsychosocial model, the patient benefits from strong family support and specialized public assistance. However, due to fall risk and progressive motor decline, wheelchair use appears imminent. This highlights the need for personalized, multidisciplinary interventions to improve gait, mobility, and functional autonomy (Bogaert et al. 2024). Additionally, limited public accessibility and ataxia‐related speech and dressing difficulties further restrict social participation, reinforcing the importance of adapted rehabilitation strategies.

These findings highlight the role of physiotherapy in improving functional impairments in SCA, particularly in dizziness and foot biomechanics. However, the limited impact on postural stability suggests the need for complementary rehabilitation strategies, such as vestibular rehabilitation and intensive functional training.

## 5 Implications for Physiotherapy Practice

This case report reinforces the importance of physiotherapy in managing SCA, demonstrating that targeted interventions can improve functional parameters such as plantar arch index and contact surface. Despite the progressive nature of SCA, physiotherapy plays a critical role in symptom management by promoting adaptive strategies that support postural control and functional mobility. These findings highlight the need for integrating vestibular rehabilitation and intensive functional training into standard physiotherapy protocols to optimize clinical outcomes. Additionally, the use of baropodometry and stabilometry in routine assessments can enhance individualized treatment planning, allowing better tracking of disease progression and therapeutic response. These insights underscore the importance of a multidisciplinary approach and long‐term rehabilitation strategies to maximize independence and quality of life in patients with SCA.

## Author Contributions

L.H.F.M. and B.R.B. drafted the manuscript. I.N.d.S.R., A.B.S.d.C., H.M.d.S.C., and B.R.B. conducted the physiotherapy interventions and data collection. L.H.F.M. and B.R.B. performed the data analysis and interpretation. S.A.S.d.M. supervised the interventions and critically revised the manuscript. All authors have read and approved the final version.

## Funding

The authors have nothing to report.

## Ethics Statement

The report was approved by the Human Research Ethics Committee of the Federal University of Pará (Protocol number: 46775421.0.0000.0018) on May 10, 2021, as determined by Resolution 510/16 of the National Health Council of Brazil.

## Consent

The patient in this case report provided written informed consent for the publication of this case report, in accordance with the ethical guidelines determined by Resolution 510/16 of the National Health Council of Brazil.

## Conflicts of Interest

The authors declare no conflicts of interest.

## Permission to Reproduce Material From Other Sources

The authors have nothing to report.

## Data Availability

The data used in this report are available from the corresponding author upon reasonable request.
